# Atypical subcutaneous nodule-type paragonimiasis in 3 children: case series

**DOI:** 10.3389/fsurg.2026.1848773

**Published:** 2026-07-16

**Authors:** Zhaokun Guo, Ying Jiang, Keheng Deng, Jun Zhou, Pan Jiao, Wenxiang Zhu, Zhongjing Zhang, Xiangyou Zhao

**Affiliations:** Department of Pediatric Surgery, The First Affiliated Hospital of China Three Gorges University (Yichang Central People's Hospital), Yichang, China

**Keywords:** diagnosis and treatment, eosinophils, pediatric paragonimiasis, praziquantel, subcutaneous nodules

## Abstract

**Objective:**

To analyze the clinical characteristics of atypical subcutaneous nodule-type paragonimiasis in children and accumulate experience in its diagnosis and treatment.

**Methods:**

A retrospective analysis was conducted on the clinical data of 3 pediatric cases with atypical subcutaneous nodule-type paragonimiasis.

**Results:**

All patients had a clear epidemiological history, significantly elevated peripheral blood eosinophils and positive *Paragonimus* antibodies. Oral praziquantel achieved excellent therapeutic effects, and surgery could serve as an adjuvant treatment.

**Conclusion:**

Paragonimiasis presents with complex and diverse clinical manifestations, and its clinical symptoms and signs lack specificity. Attention should be paid to taking a detailed epidemiological history; elevated eosinophils and positive *Paragonimus* antibodies are helpful for diagnosis. Oral praziquantel combined with adjuvant surgical treatment can yield good clinical outcomes.

## 1 Introduction

Paragonimiasis is a globally distributed food-borne zoonotic parasitic disease caused by more than 50 recognized species of *Paragonimus* and eight can infect humans (1). In China, *P. westermani* (2), *P. skrjabini* (3, 4) and *P. heterotremus* (5, 6) are the most prevalent and clinically significant human pathogens. Paragonimiasis is a food-borne zoonotic parasitic disease (7). Human infection primarily occurs through consumption of raw or undercooked freshwater crabs (8, 9) and crayfish harboring metacercariae (10, 11), or ingestion of raw water contaminated with these infective larvae (12–14). Clinical manifestations of paragonimiasis are highly variable and depend on the site of infection, which can include the lungs (15, 16), abdomen (17), subcutaneous tissue (18), and central nervous system (19). Subcutaneous nodule-type paragonimiasis, the most common extrapulmonary form in China, is particularly prone to misdiagnosis due to its non-specific clinical features, which can easily be confused with lipoma (20), eosinophilic granulomatosis with polyangiitis (EGPA) (21), post-COVID-19 condition (22), pneumonia, pulmonary tuberculosis, or lung cancer (23) and other soft tissue lesions, resulting in a high clinical misdiagnosis rate. Although paragonimiasis is classified as a neglected tropical disease (NTD) for the 2014–2015 nationwide parasitological survey documented a crude prevalence rate of only 0.005% in China (23), the incidence of paragonimiasis has shown an increasing trend in the Three Gorges Reservoir region (24) and adjacent areas (25). Herein, we report the clinical data of 3 pediatric patients with atypical subcutaneous nodule-type paragonimiasis admitted to our department between October 2024 and March 2025, aiming to improve the early recognition and accurate diagnosis of this rare clinical entity.

## 2 Materials and methods

### 2.1 General information

Among the 3 pediatric patients, there were 2 boys and 1 girl, aged 8 years and 10 months, 5 years and 4 months, and 6 years and 3 months, respectively. The masses were located in the left abdominal wall, left waist, and right inguinal region, respectively. Physical examination findings were as follows: 1. A hard mass with a size of approximately 5.0 cm × 3.0 cm was visible in the left lower abdominal wall, with unclear boundaries, slightly erythematous local skin, tenderness, and no obvious fluctuant sensation. 2. A single mass with a size of about 3.0 cm × 3.0 cm was palpable in the left lumbodorsal region, with no skin erythema on the surface, hard texture, tenderness and mild pruritus, and poor mobility. 3. A mass with a range of about 5 cm × 10 cm was palpable in the right inguinal region, accompanied by soft tissue thickening and edema, unclear boundaries, non-erythematous skin, normal skin temperature, no tenderness and no fluctuant sensation. All 3 patients were admitted due to subcutaneous masses. One patient had pleural effusion on admission, one had pleural effusion 3 months prior to admission, and one had peritoneal effusion (more than 1 month prior to admission). Fine-needle aspiration cytology was performed in 1 case, and surgical resection with pathological biopsy was conducted in 2 cases (Table 1).

**Table 1:** The clinical data of three pediatric patients.

No.	Sex	Age	Chief complaint	Mass location	Specialized physical examination	Accompanying symptoms	Special past history	Pathological examination	Pathological no.	Surgery	Follow-up
1	Male	8 years and 10 months	Left lower abdominal wall mass found for 1 week	Left abdominal wall	A hard mass of about 5.0 cm × 3.0 cm was palpable, with unclear boundary, slight redness and swelling of local skin, tenderness, and no obvious fluctuation.	Pleural effusion	History of playing in streams	1. Fine needle aspiration cytology of abdominal wall mass; 2. Surgical resection of abdominal wall mass for pathological examination	Needle aspiration cytology: D2400688; Routine pathology: 2415996	Yes	No recurrence during 6-month follow-up
2	Male	5 years and 4 months	Left lumbar mass found for more than 1 month	Left lumbar region	A mass of about 3.0 cm × 3.0 cm was palpable, no redness and swelling on the surface, hard texture, tenderness and slight itching, poor mobility	None	Diagnosed with “paragonimiasis” due to pleural effusion 3 months ago	Pathological examination after surgical resection	Routine pathology: 2601185	Yes	No recurrence during 1-month follow-up
3	Female	6 years and 3 months	Right inguinal mass found for 2 weeks	Right inguinal region	A mass of about 5 cm × 10 cm was palpable, accompanied by thickening and edema of soft tissue, unclear boundary, no redness of skin, normal skin temperature, no tenderness, no fluctuation	None	Abdominal effusion was found during laparoscopic high ligation of bilateral indirect inguinal hernia sac 1 month ago; history of playing in streams	None	None	No	No recurrence during 1-month follow-up

### 2.2 Auxiliary examinations

#### 2.2.1 Routine blood test

Eosinophil percentages were 24.6%, 44.5% and 31.8% in patient 1, patient 2 and patient 3, respectively.

#### 2.2.2 Serological test for parasitic pathogens (7 items)

All 3 patients had positive *Paragonimus* IgG antibodies (+) (samples sent to external laboratory for detection).

#### 2.2.3 The color Doppler ultrasonography (US) of soft tissue masses were as follows

**Figure 1:**
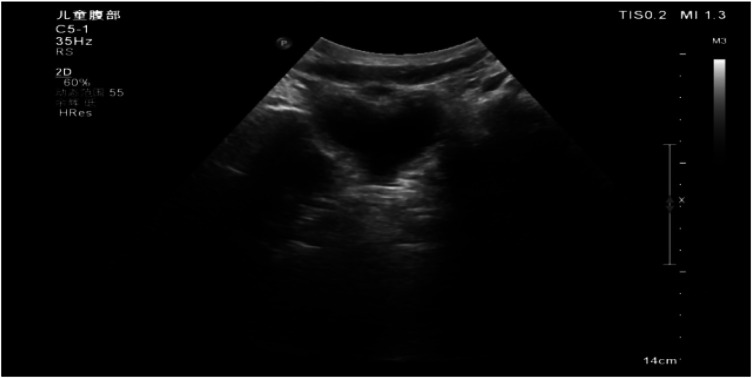
A slightly hyperechoic mass with a size of approximately 4.8 cm × 2.0 cm was seen in the subcutaneous tissue, with no obvious capsule and heterogeneous internal echo. Color flow imaging (CDFI) showed blood flow signals within the mass. **Impression**: Suspected lipoma (Patient 1).

**Figure 2:**
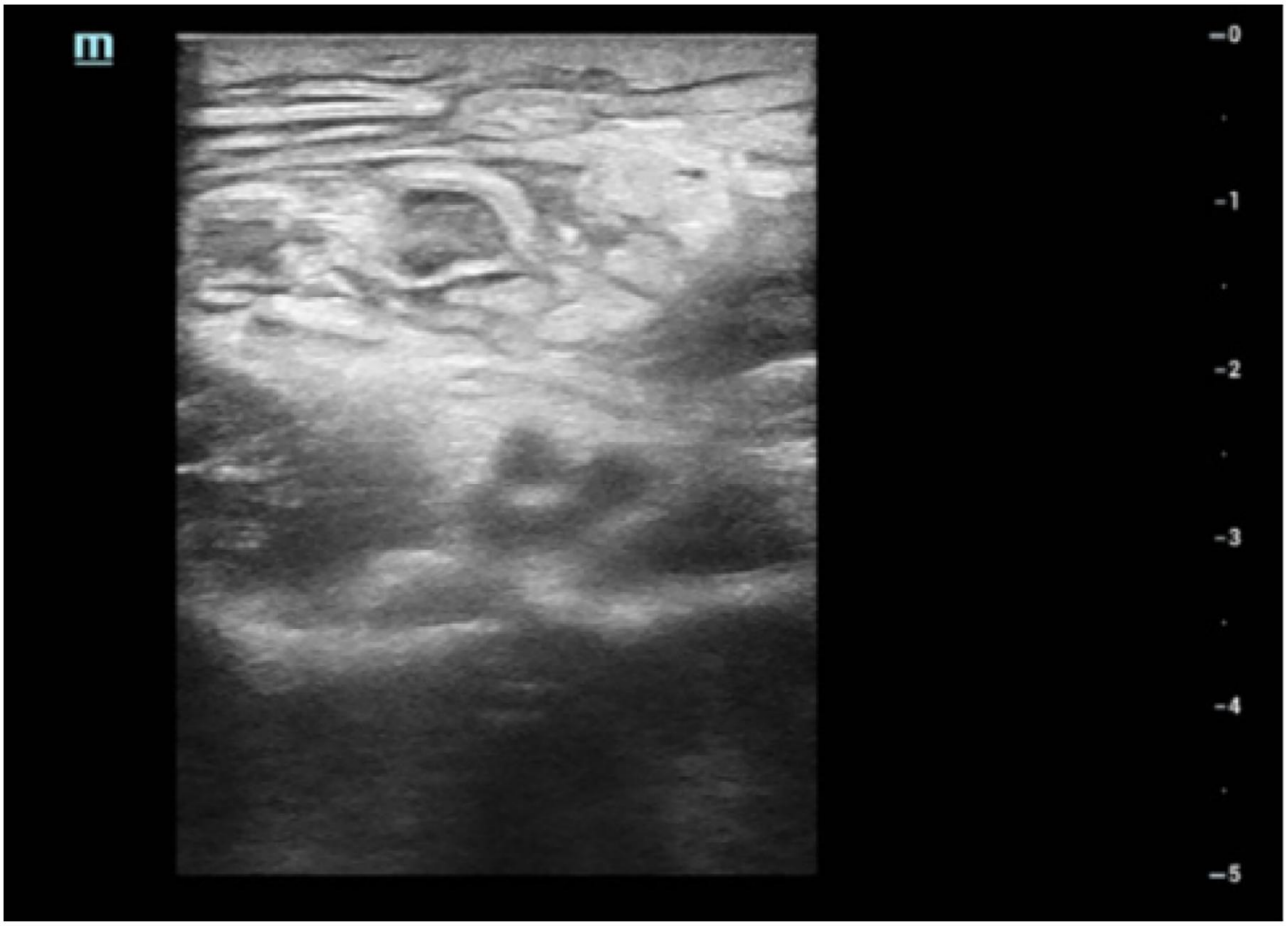
Subcutaneous soft tissue thickening, edema and multiple lymph nodes were found in the right inguinal region. **Impression**: Suspected inflammatory changes (Patient 2).

**Figure 3:**
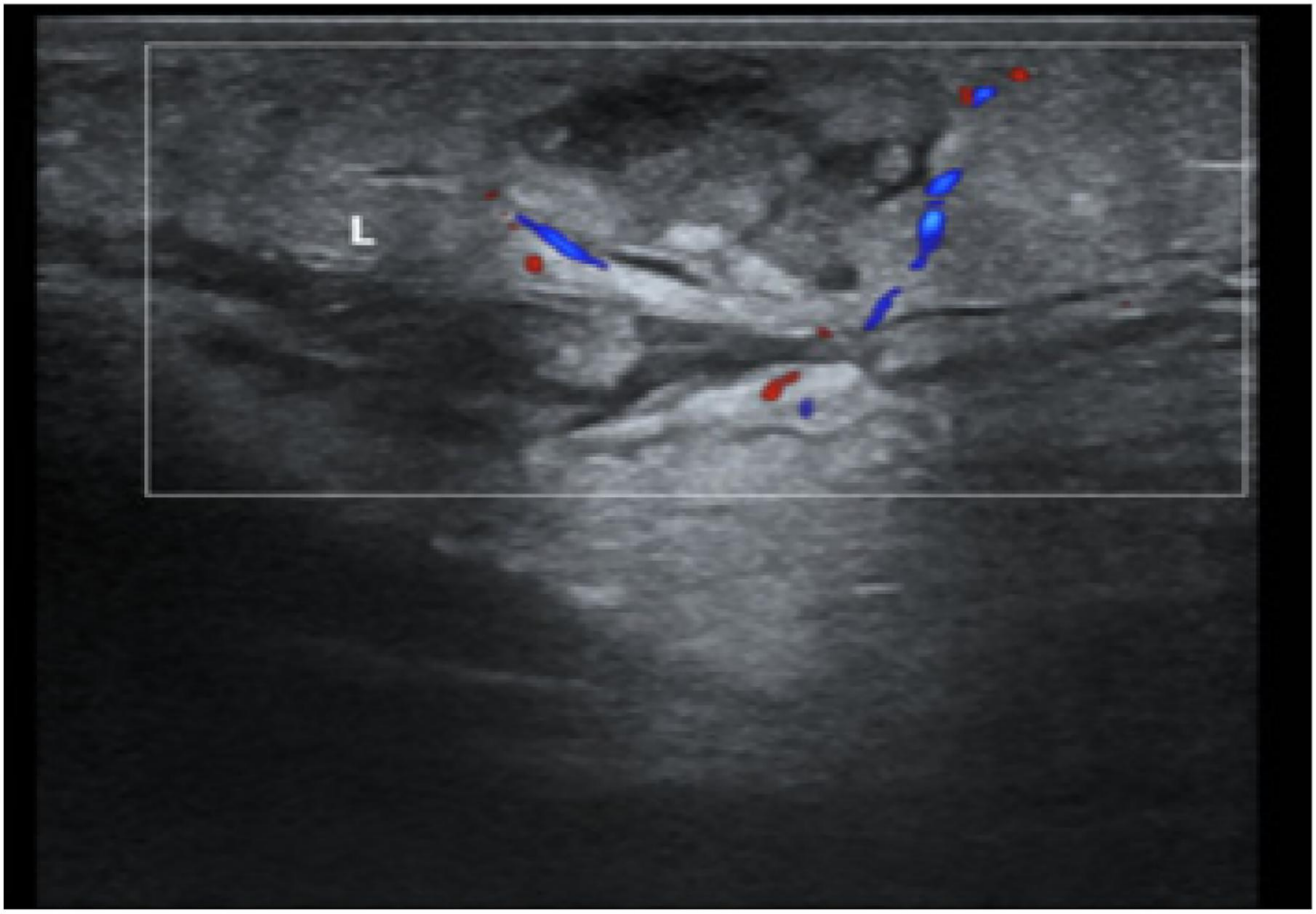
Subcutaneous soft tissue edema with irregular anechoic areas was observed in the left lumbodorsal region. **Impression**: Suspected inflammatory changes, clinical correlation recommended (Patient 3).

All three patients were found to have solid masses [Figures 1–3].

#### 2.2.4 Chest computed tomography (CT)

**Figure 4:**
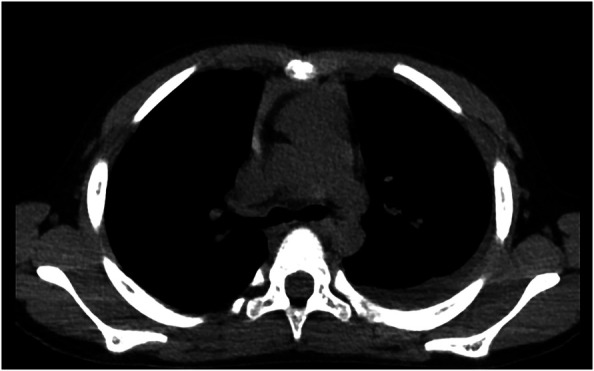
No obvious active lesions were found in both lungs. Mixed density shadows were seen in the left thoracic cavity, suggesting pleural lesions complicated with effusion? All main bronchi were unobstructed, and mediastinal lymph nodes were not enlarged (Patient 1).

Patient 1 was found to have pleural effusion [Figure 4].

#### 2.2.5 Pathological findings

**Figure 5:**
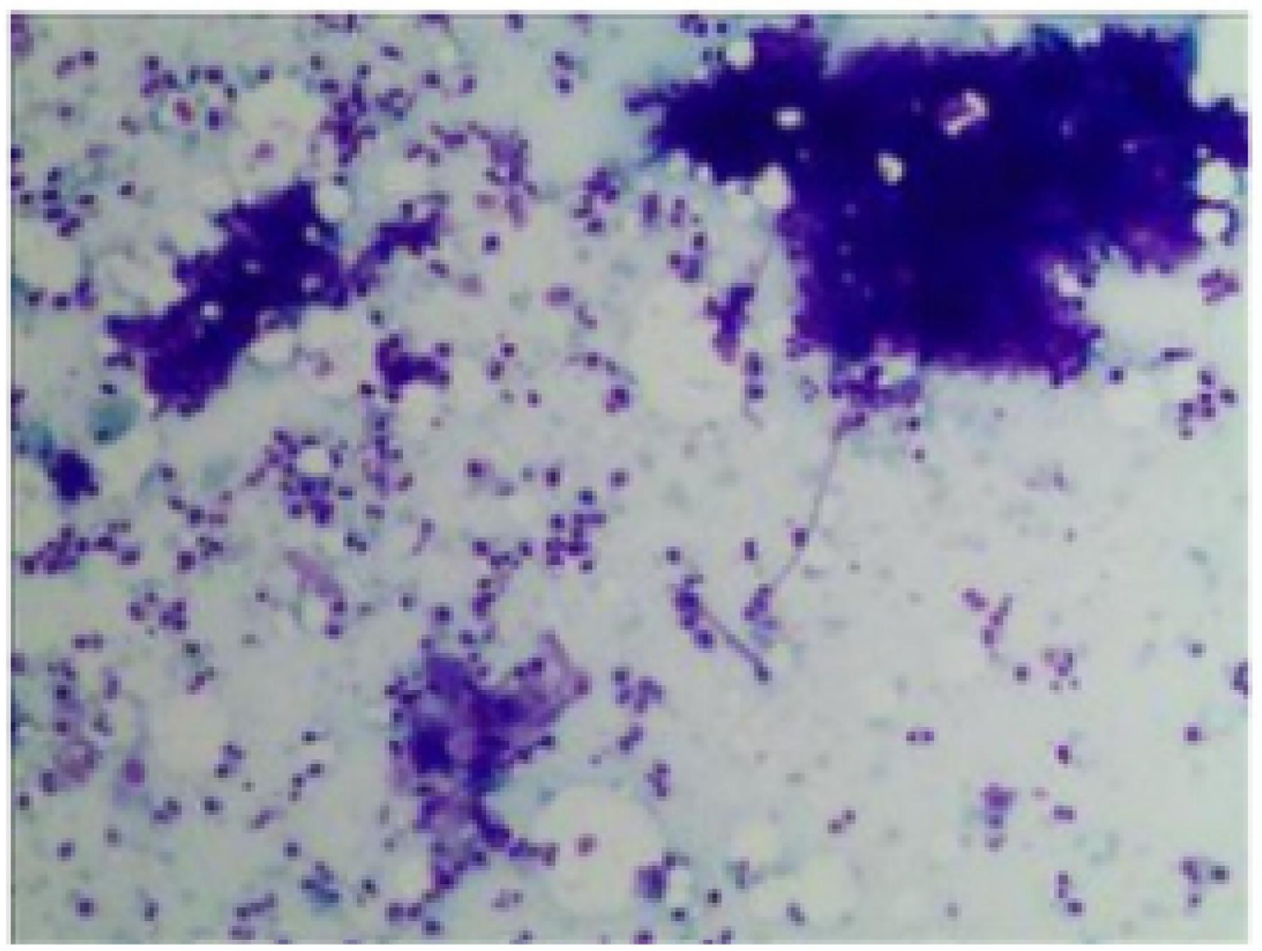
Aspiration smear and staining of the left lower abdominal wall mass: under light microscopy, a large number of degenerative necrotic materials, eosinophils, a small number of neutrophils, lymphocytes, macrophages and occasional multinucleated giant cells were seen (Patient 1).

**Figure 6:**
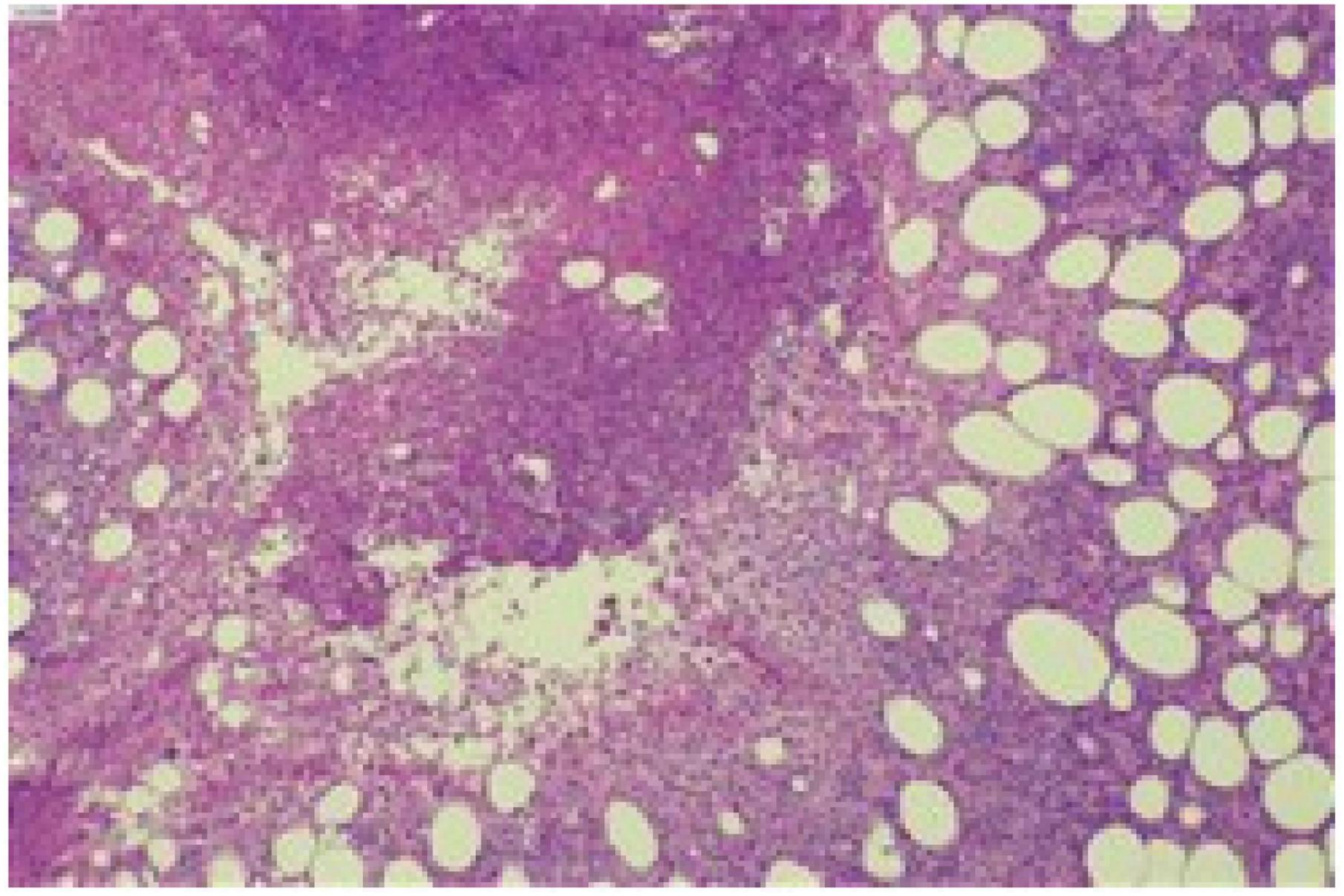
Pathological examination of the abdominal wall mass: multiple sinus-like and tunnel-like clefts (diameter about 0.15 cm) were found in the subcutaneous fibrous connective tissue under microscopy, with a small amount of epithelioid cell granulomas formed around them and extensive eosinophilic infiltration. No other abnormal findings were observed. **Conclusion**: The abdominal wall lesion in this case is considered to be caused by parasitic migration and burrowing. However, no parasitic worms were found in the lesion, and it cannot be ruled out that the worms have migrated to distant sites. Comprehensive analysis combined with clinical data is recommended (Patient 1).

**Figure 7:**
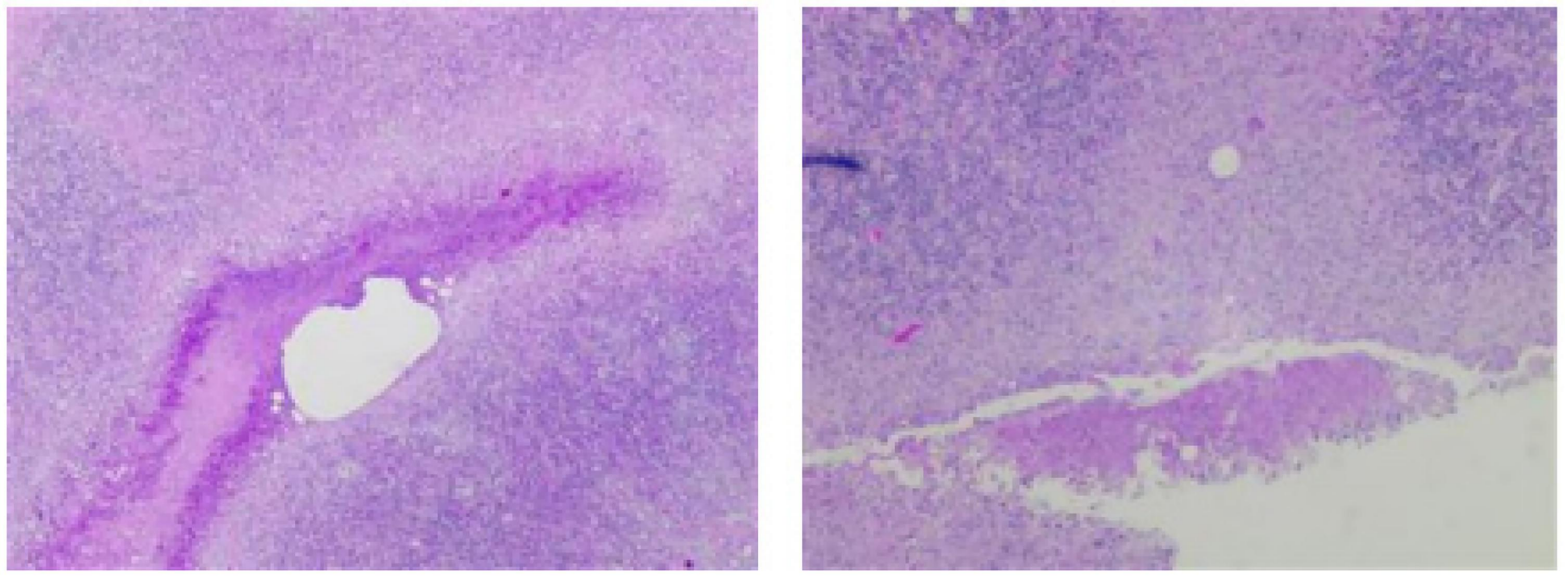
Pathological examination of the left psoas major muscle mass: microscopically, it showed well-differentiated fibroadipose tissue with focal necrosis. Histiocytes were seen around the necrotic area, and extensive inflammatory cell infiltration dominated by eosinophils and lymphocytes was found in the interlobular and septal areas of adipose lobules. The morphological features were consistent with eosinophilic panniculitis, and parasitic infection cannot be excluded. Comprehensive analysis combined with serological test results is recommended by clinicians (Patient 2).

The pathological results of patients 1 and 2 indicated the presence of eosinophilic cell aggregation, and patient 2 was found to have a tunnel caused by parasites [Figures 5–7].

### 2.3 Surgical methods

Two children underwent surgical resection. Case 1 received resection of the abdominal wall mass, and Case 2 underwent resection of the lumbar mass. Intraoperatively, both masses were located subcutaneously, closely attached to the skin without capsules. The lesions were fragile with ill-defined borders from the surrounding tissues. All masses were completely excised and sent for pathological examination. The pathology number of Case 1 is 2415996, and that of Case 2 is 2601185.

## 3 Results

All children received praziquantel for anthelmintic treatment after definite clinical diagnosis. The dosage regimen was as follows: praziquantel 25 mg/kg per dose, administered three times daily for 3 consecutive days as one course. A second course with the same dosage was given after a 1-week interval. A total of 3 courses of anthelmintic therapy were completed. Clinical cure was defined as complete resolution of symptoms and normal eosinophil count.

Patient 1 was admitted on October 19, 2024. After comprehensive examinations and multidisciplinary discussion, resection of the left abdominal wall mass was performed under general anesthesia on October 25, 2024. Pathological reports were released on October 28, 2024. Combined with laboratory findings, *Paragonimus* was diagnosed. Pediatric consultation was arranged and anthelmintic treatment was recommended. The patient completed 3 courses of anthelmintic therapy after discharge, with all clinical symptoms relieved and laboratory indicators returning to normal. No recurrence was observed during the 6-month follow-up.

Three months prior to admission, Patient 2 was diagnosed with *Paragonimus* in the pediatric department due to pleural effusion and recovered after 3 courses of anthelmintic treatment. One month after drug discontinuation, a lumbar mass was noticed and gradually enlarged. The patient was then admitted on January 27, 2026. Resection of the lumbar muscular and soft tissue mass was performed under general anesthesia on January 29, 2026. One course of anthelmintic therapy was given postoperatively. Clinical symptoms subsided and all laboratory results were normal, with no recurrence during the 1-month follow-up.

One month before admission, peritoneal effusion was detected during laparoscopic high ligation of hernia sacs in Patient 3, while routine and biochemical tests of ascites showed no abnormalities. The patient was admitted on January 8, 2026. Parasite tests and blood next-generation sequencing (NGS) were completed. A pediatric consultation on January 13, 2026 confirmed *Paragonimus* and anthelmintic treatment was suggested. After 3 courses of treatment, clinical symptoms disappeared and laboratory findings normalized. No recurrence was noted in the 1-month follow-up.

## 4 Discussion

The subcutaneous nodule-type paragonimiasis is a relatively rare extrapulmonary form that ranks in incidence after the thoracic-pulmonary and abdominal types (5). The core cause of subcutaneous nodules induced by paragonimiasis is the migration and parasitism of *Paragonimus* larvae in the subcutaneous tissue. Meanwhile, mechanical damage caused by worm migration superimposed with inflammation induced by the worms and their metabolites can lead to lymphatic reflux obstruction and the formation of pleuroperitoneal exudate dominated by eosinophils (6). The typical nodules of this disease present as painless subcutaneous masses, which can be single or multiple, variable in size, with clear boundaries, good mobility and no adhesion to the skin. Some patients may have mild pruritus but no erythema, ulceration or exudation, which is consistent with the inguinal mass in patient 2 in this case series. A few patients may have tender nodules, as seen in patient 1 and patient 3 in this study. Nodules caused by *Paragonimus skrjabini* infection have characteristic migratory features and may appear successively in different parts of the body, serving as an important clue for differential diagnosis. Subcutaneous nodular paragonimiasis does not exist in isolation; it can occur alone or as a mixed type combined with thoracic-pulmonary or abdominal types (13). Approximately 10%–30% of patients are complicated with pleuroperitoneal effusion, and among the 3 children in this study, 2 had pleural effusion (patient 1 and patient 2) and 1 had peritoneal effusion (patient 3).

In terms of auxiliary examinations, significantly elevated peripheral blood eosinophils are the most characteristic manifestation of this disease (26, 27). The eosinophil percentages of the 3 children in this study on admission were 24.6%, 44.5% and 31.8% respectively. Even in the absence of subcutaneous nodules, unexplained eosinophilia should alert clinicians to the possibility of parasitic infection (28). *Paragonimus* antibody detection is the key to definite diagnosis. Enzyme-linked immunosorbent assay (ELISA) is the most commonly used method in clinical practice, with a positive rate of 83.33%–90% but certain cross-reactivity, while dot immuno-gold filtration assay (DIGFA) has a positive rate of up to 100%, is simple and rapid to operate, and is more suitable for clinical emergency diagnosis (29). Chest CT has important indicative value for patients with combined thoracopulmonary involvement, with typical manifestations including multiple nodules, patchy opacities, cavities and pleural effusion, while B-ultrasound of subcutaneous nodules has no specific manifestations and limited diagnostic value (9). Pathological examination is the gold standard for diagnosis, with typical manifestations of extensive eosinophilic infiltration, and eosinophilic granulomas and Charcot-Leyden crystals may be seen in some cases. Definite diagnosis can be made if worms or eggs are found, but the clinical detection rate is relatively low. Only eosinophilic infiltration was seen in the 2 surgically treated cases in this study (Patient 1 and 2), and only 1 case had worm-like sinus tracts (Patient 1). Therefore, a negative pathological examination cannot rule out the diagnosis of paragonimiasis, and comprehensive judgment combined with antibody detection and other indicators is required.

The diagnosis of this disease should follow the principle of comprehensive judgment, combining epidemiological history, clinical manifestations and various auxiliary examinations for a full evaluation. Epidemiological history is the premise of diagnosis, and a history of eating raw freshwater crabs or crayfish or contact with contaminated water in endemic areas is the most important clue. Patients 1 and 3 had a history of playing in streams. Since children are usually unable to clearly describe their medical histories, clinicians should carefully inquire about their living habits for pediatric patients in endemic areas presenting with unexplained subcutaneous nodules and eosinophilia (26, 27). Painless, mobile subcutaneous nodules with significantly elevated peripheral blood eosinophils are the core clinical clues, positive *paragonimus* antibody is the most commonly used diagnostic basis in clinical practice, and pathological discovery of worms or eggs is the final diagnostic basis.

The treatment principle is anthelmintic treatment as the mainstay and surgical treatment as an adjunct, and standardized praziquantel regimens can achieve good clinical efficacy. As the first-line drug for the treatment of paragonimiasis, praziquantel has the characteristics of convenient oral administration, short course of treatment and mild adverse reactions (30). The conventional regimen is 25 mg/kg per dose, three times a day, for a 3-day course, repeated with a 1-week interval until clinical cure. In this case series, the subcutaneous nodules of patient 3 completely subsided and eosinophil counts returned to normal after 3 courses of praziquantel treatment; patient 1 had normalized eosinophil counts after 3 courses of praziquantel treatment following surgical resection; patient 2 had received 3 courses of praziquantel treatment before surgical resection but developed a lumbodorsal mass 2 months after drug withdrawal, and achieved normalizeikd eosinophil counts after surgical resection plus an additional course of praziquantel treatment. These findings suggest that a full course of standardized medication is the key to successful treatment, and surgery can assist in diagnosis and treatment. Some patients may experience nodule enlargement in the early stage of treatment, but the continuous decrease of eosinophil counts is an indicator of effective treatment, and drug withdrawal is not required. Surgical treatment is mainly applicable for pathological examination and can also assist in relieving symptoms, but it cannot replace anthelmintic treatment. Standard oral administration of praziquantel is still required after surgery to avoid disease recurrence due to incomplete eradication of worm bodies. For patients with mixed type complicated with pleural effusion or pericardial effusion, symptomatic drainage treatment can be given simultaneously with anthelmintic treatment to improve therapeutic effects.

In summary, subcutaneous nodular paragonimiasis has obvious epidemiological characteristics in endemic areas. Painless, mobile subcutaneous nodules accompanied by significantly elevated peripheral blood eosinophils are the core clinical clues, and antibody detection and pathological examination are the keys to definite diagnosis. For patients with unexplained subcutaneous nodules in endemic areas, particularly children, clinicians should obtain a detailed epidemiological history and promptly perform appropriate diagnostic workup to facilitate early diagnosis, while avoiding diagnostic bias due to excessive reliance on isolated test results or focal symptoms. After diagnosis, standardized praziquantel treatment should be given and full-course medication should be ensured. At the same time, it is necessary to strengthen the training of clinicians and health education for residents in endemic areas, improve disease awareness and vigilance, and reduce the harm of the disease from both diagnosis and prevention aspects.

## Data Availability

The raw data supporting the conclusions of this article will be made available by the authors, without undue reservation.
